# Effect of tidal volume and positive end‐expiratory pressure on expiratory time constants in experimental lung injury

**DOI:** 10.14814/phy2.12737

**Published:** 2016-03-20

**Authors:** William R. Henderson, Paolo B. Dominelli, Yannick Molgat‐Seon, Rachel Lipson, Donald E. G. Griesdale, Mypinder Sekhon, Najib Ayas, A. William Sheel

**Affiliations:** ^1^ Division of Critical Care MedicineDepartment of MedicineFaculty of MedicineUniversity of British ColumbiaVancouverBritish ColumbiaCanada; ^2^ School of KinesiologyUniversity of British ColumbiaVancouverBritish ColumbiaCanada; ^3^ Emmes CanadaBurnabyBritish ColumbiaCanada; ^4^ Department of Anesthesiology, Pharmacology & TherapeuticsUniversity of British ColumbiaVancouverBritish ColumbiaCanada

**Keywords:** Acute respiratory distress syndrome, pulmonary alveoli, respiration

## Abstract

We utilized a multicompartment model to describe the effects of changes in tidal volume (*V*_T_) and positive end‐expiratory pressure (PEEP) on lung emptying during passive deflation before and after experimental lung injury. Expiratory time constants (*τ*_E_) were determined by partitioning the expiratory flow–volume ($\dot{V}$ _E_V) curve into multiple discrete segments and individually calculating *τ*_E_ for each segment. Under all conditions of PEEP and *V*_T_,* τ*_E_ increased throughout expiration both before and after injury. Segmented *τ*_E_ values increased throughout expiration with a slope that was different than zero (*P *<* *0. 01). On average, *τ*_E_ increased by 45.08 msec per segment. When an interaction between injury status and *τ*_E_ segment was included in the model, it was significant (*P *<* *0.05), indicating that later segments had higher *τ*_E_ values post injury than early *τ*_E_ segments. Higher PEEP and *V*_T_ values were associated with higher *τ*_E_ values. No evidence was found for an interaction between injury status and *V*_T_, or PEEP. The current experiment confirms previous observations that *τ*_E_ values are smaller in subjects with injured lungs when compared to controls. We are the first to demonstrate changes in the pattern of *τ*_E_ before and after injury when examined with a multiple compartment model. Finally, increases in PEEP or *V*_T_ increased *τ*_E_ throughout expiration, but did not appear to have effects that differed between the uninjured and injured state.

## Introduction

Acute respiratory distress syndrome (ARDS) is a lung injury characterized by hypoxia and impaired pulmonary mechanics. The associated histological changes, such as alveolar flooding and collapse, airway edema, and altered surfactant function, are heterogeneous in distribution within the lungs (Dakin et al. 2011). Because of this heterogeneity, some lung units have abnormal values of resistance (*R*) and/or elastance (*E*), while others have values that are similar to those found in healthy lung (Eissa et al. 1991b; Schiller et al. 2001; Otto et al. 2008; Mertens et al. 2009; Kaczka et al. 2011a,b). The *R* and *E* of a lung unit determine its time constant (*tau* [*τ*]), which is defined as the time required to inflate or deflate 63% of the volume of a given lung unit. The regional variation in *R* and *E* of lung tissue implies that there is regional heterogeneity in expiratory time constants (*τ*_E_).

Currently, there is scant information regarding the extent to which the pattern of *τ*_E_ is altered by the development of lung injury in a given subject. We have recently demonstrated that the *τ*_E_ pattern of lung passive expiration in the same subject differs with injury status and that these patterns can be altered by manipulating the density of the ventilating gas (Henderson et al. 2015). Kondili and colleagues have examined changes in *τ*_E_ due to manipulation of positive end‐expiratory pressure (PEEP) following lung injury (Kondili et al. 2002). They found that in patients with ARDS at zero PEEP, *τ*_E_ increased throughout expiration due to progressive increases in respiratory system resistance (*R*_RS_). The application of PEEP decreased *R*_RS_ (primarily in late expiration), resulting in *τ*_E_ values that were smaller and less varied throughout lung emptying. Similarly, Kondili and colleagues found that the addition of PEEP increased respiratory system elastance (*E*_RS_) and *τ*_E_ during the early portion of expiration.

No studies have documented the role of altering PEEP or tidal volume (*V*_T_) on the pattern of *τ*_E_ in the same subject before and after injury. This is relevant, as a more complete understanding of how PEEP and *V*_T_ interact with injury status may alter mechanical ventilation strategies. For example, the optimal combination of PEEP and *V*_T_ may differ in the same patient as lung injury evolves and resolves. Recognition of these changes may allow clinicians to optimize the pattern of lung emptying and minimize the risk of new ventilator‐induced lung injury.

We reasoned that interventions that alter regional *E* and *R*, such as alterations in *V*_T_ or PEEP, should alter *τ*_E_ values and the pattern or passive expiration. We hypothesized that the effects of both PEEP and *V*_T_ on *τ*_E_ would differ between the uninjured and injured states. To this end, we undertook to characterize the effects of changes in PEEP and *V*_T_ on *τ*_E_, *R*_RS_, and *E*_RS_ before and after the induction of an experimental model of lung injury.

## Methods

### Animals and instrumentation

The Animal Research Committee of the University of British Columbia (certificate #: A12‐0272) reviewed and approved the experimental procedures. Anesthesia was induced with inhaled isoflurane (3–5% in oxygen) after sedation with telazol (4–6 mg/kg intramuscular injection) in six adult female Yorkshire X pigs (weight, 31.42 ± 5.42 kg). After tracheal intubation, inhalational anesthesia was discontinued once total intravenous anesthesia was established with midazolam (0.1 mg/kg intravenous) and a propofol infusion (200 *μ*g/kg/min and adjusted to between 150 and 300 *μ*g/kg/min). The adequacy of anesthesia was assessed every 15 min using assessment of vital signs, physical examination, and electrocardiography. The animals were mechanically ventilated (Puritan‐Bennett 7200, Covidien, Ireland) with 0 cm H_2_O of PEEP using an inspired oxygen fraction (FiO_2_) of 0.5 and *V*_T_ of 10 cc/kg. Breathing frequency was initially set at 15 breaths/min, and was adjusted to maintain end‐tidal CO_2_ between 35 and 45 mmHg with an inspiratory flow of 45 L/min. A right femoral artery catheter was used to collect arterial blood samples into preheparinized syringes, which were immediately analyzed by calibrated blood gas analyzer (ABL 80 CO‐OX Flex). Neuromuscular blockade was induced when needed prior to all measurements of pulmonary mechanical parameters using pancuronium (0.05–0.1 mg/kg intravenous) after a bolus of intravenous midazolam (0.1 mg/kg). Paralysis was monitored by assessment of response to train‐of‐four stimulation using a peripheral nerve stimulator on the palmar side of the forelimb. At the end of the experiment, euthanasia was achieved with pentobarbital sodium (120 mg/kg intravenous). Death was confirmed by the absence of a pulse and cardiac electrical activity on continuous surface electrocardiography.

### Induction of lung injury

We used a previously published method (Henderson et al. 2014) of lung injury that satisfies the current American Thoracic Society's guidelines for a high‐quality model of ARDS and that demonstrates a profound neutrophilic alveolitis with diffuse alveolar damage (Matute‐Bello et al. 2011). Sodium polyacrylate gel (1%) in aqueous solution was injected through the endotracheal tube and was manually dispersed throughout the lungs by bagging. One 5‐mL aliquot was given every 5 min until an arterial oxygen tension (PaO_2_) of less than 150 mmHg, while receiving a fraction of inspired oxygen (FiO_2_) of 0.5 was observed. The animals required 3 ± 1.4 h after injury to achieve this degree of hypoxia. The ratio of PaO_2_/FiO_2_ less than 300 was chosen to be consistent with current definitions of ARDS (Ranieri et al. 2012).

### Interventions

Prior to and subsequent to experimental lung injury, animals were ventilated in a computer‐generated random order with six different combinations of *V*_T_ and PEEP: *V*_T_ of 5, 10, 12, and 15 cc/kg all at 0 cm H_2_O PEEP along with 5 and 10 cm H_2_O PEEP at 12 cc/kg. PEEP and *V*_T_ levels were chosen to allow the assessment of a range of clinically relevant PEEP and *V*_T_ values and reflect those used in recent similar studies (Pelosi et al. 2001; Kondili et al. 2002). Prior to each set of measurements, the animals were ventilated for 20 min at each combination of *V*_T_ and PEEP to eliminate the effect of volume history.

### Measurement of pulmonary mechanics

All data were collected and recorded digitally (PowerLab/16SP model ML 795 and Chart v7, ADI, Colorado Springs, CO). Data sampling occurred at a frequency of 1000 Hz. Using heated pneumotachographs (Model 3813, Hans Rudolph, Kansas City, MO), inspiratory and expiratory flows ($\dot{V}$_I_ and $\dot{V}$_E_) were measured and subsequently integrated using a trapezoidal technique to determine inspiratory and expiratory volumes (*V*_I_ and *V*_E_). Each analog input was fitted with a fixed 25‐kHz low‐pass filter which functions as the antialiasing feature.

Airway pressure (*P*_AW_) was measured at a port distal to the ventilator wye and pleural pressure was assumed to be approximated by the measurement of esophageal pressure (*P*_ES_) with a balloon‐tipped catheter (Ackrad Laboratory, Cranford, NJ). The catheter was positioned in the lower third of the esophagus and balloon position was verified by the presence of cardiac pulsation in the trace and the adequacy of waveform shape during mechanical ventilation (Baydur et al. 1982; Talmor et al. 2008). PVC pressure tubing (2 mm internal diameter, 3 mm outer diameter) with male and female Luer lock connections were used to connect all apparatus. *P*_AW_ and *P*_ES_ were referenced to atmospheric pressure and measured using calibrated pressure transducers (Raytech Instruments, Vancouver, BC, Canada).

Tracheal pressure (*P*_TR_) is often assumed to be estimated by *P*_AW_. This assumption may not be valid under dynamic conditions such as when flow‐ dependent resistance across the endotracheal tube creates a time‐dependent pressure drop across the endotracheal tube (*P*_ETTV(t)_). The drop in pressure causes *P*_TR_ to differ significantly from *P*_AW_ (Uchiyama et al. 2009)_._ To overcome this, *P*_TR_ at a specific time (*P*_TRV(t)_) may be measured directly or calculated at any time point given that (*P*_TRV(t)_) = *P*_AW(t)_ − (*P*_ETTV(t)_) (Guttmann et al. 1993)_._ To calculate *P*_TRV(t)_, we used a previously validated multifactor formula that estimates the pressure drop across the endotracheal tube from three known values: the endotracheal tube length and diameter, ˙ $\dot{V}$_E_ and *P*_AW_ (Guttmann et al. 1993, 1995). The model is defined as: *P*_TRV(t)_ = *P*_AW_(t) − *K*_1_ $\dot{V}$ ^K2^, where *K*_1_ and *K*_2_ are empirically derived values from previous work (Guttmann et al. 1993).

Elastance of the respiratory system (*E*_RS_), that is, without the interposition of the endotracheal tube or ventilator apparatus for the entire expiration, was calculated as **Δ** *P*_TR_/**Δ** *V*. Lung elastance (*E*_L_) was calculated as Δ(*P*_TR_ − *P*_ES)_/Δ*V*. Chest wall elastance (*E*_CW_) was calculated as Δ*P*_ES_/Δ*V*. Descriptive *E*_RS_, *E*_L_, and *E*_CW_ data (as opposed to that used to calculate *τ*_E_) were collected during end‐inspiratory plateau conditions. As the flow‐dependent resistance of the endotracheal tube and ventilator apparatus alters values of *R* measured distal to the endotracheal tube (Wright and Bernard 1989; Guttmann et al. 1993), we calculated the resistance of the respiratory system without the effect of the endotracheal tube and ventilator (*R*_RS_) as Δ*P*_TR_/Δ $\dot{V}$_E_. Transpulmonary pressure (*P*_TP_) was defined as *P*_TR_ − *P*_ES_ and lung resistance (*R*_L_) was calculated as Δ*P*_TP_/Δ$\dot{V}$_E_. Chest wall resistance (*R*_CW_) was calculated as Δ*P*_ES_/Δ$\dot{V}$_E_. Functional residual capacity (FRC) was measured before and after injury using a previously described helium dilution method (Patroniti et al. 2004). Arterial blood gas analysis was performed prior to injury, after injury, and at the end of the experimental session.

### Calculation of expiratory time constants

Calculating a single value for *τ*_E_ assumes that all lung units inflate and deflate as a single compartment and does not allow differentiation between fast and slow filling/emptying units (Mcilroy et al. 1963; Brunner et al. 1995; Aerts et al. 1999). The assumption that a single all lung units have identical *τ*_E_ may have important consequences; heterogeneous rates of alveolar filling/emptying can create localized areas of high tissue strain, which may cause new lung injury and/or exacerbate existing injury (Protti et al. 2011). To address this issue, we and others have utilized a multicompartment model to describe lung emptying during passive deflation by partitioning the expiratory flow–volume ($\dot{V}$_E_V) curve into multiple discrete segments and individually calculating *τ*_E_ for each these segments (Guttmann et al. 1995; Lourens et al. 2000; Kondili et al. 2002, 2004; Henderson et al. 2015). The multisegment method allows a more nuanced description of the changes in *τ*_E_ throughout expiration, and therefore facilitates better understanding of the physiology of passive expiration than does a single‐compartment model.

**Figure 1:**
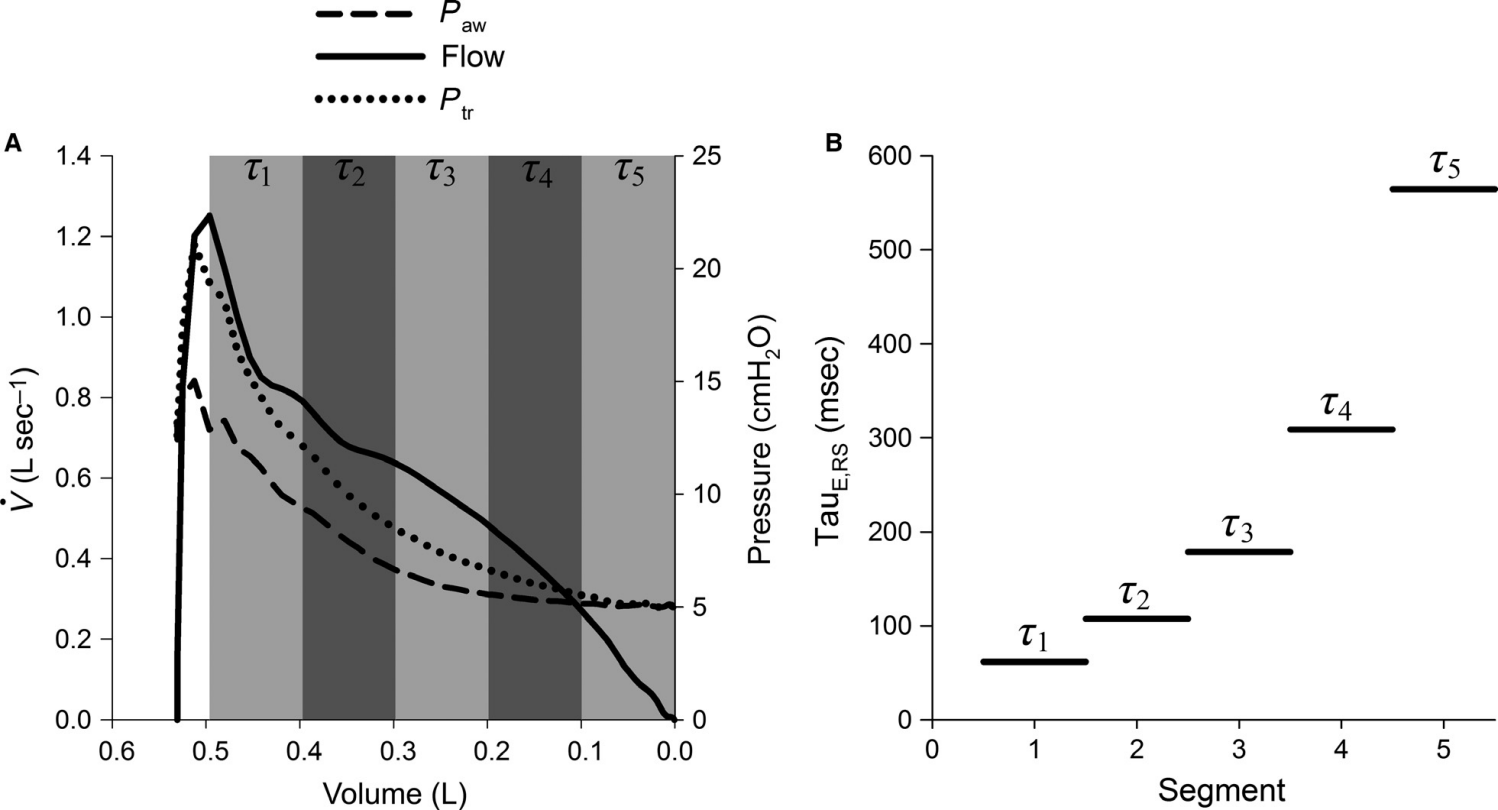
Graphical representation of the method used to derive segmental expiratory time constants (*τ*_E_) values. (A) Flow–volume ($\dot{V}$V) curve (black line), a pressure–volume (PV) curve using pressures measured at the ventilator wye and including the effects of the endotracheal tube and ventilator apparatus (dashed line), and a PV curve using tracheal pressures excluding the effects of the endotracheal tube and ventilator apparatus (dotted line) were created for each animal. Each ensemble from maximum expiratory flow to end expiration was divided into five equivolumetric segments. (B) For each segment the expiratory $\dot{V}$V curve was replaced by a least squares fitted straight line. (C) The slope of each segment provides the *τ*_E_ for that segment. In this figure, only the expiratory time constant of the respiratory system (*τ*_E_, _RS_) is displayed.

We combined 10 individual $\dot{V}$_E_V and pressure–volume (PV) traces taken at the end of 20 min of ventilation at each PEEP and *V*_T_ combination using methods described by Guttmann (Guttmann et al. 1995) and Kondili (Kondili et al. 2002). From these data, we created ensemble $\dot{V}$_E_V and PV curves for each animal for all combinations of injury state, each combination of *V*_T_ and PEEP. From these data, we calculated values for *τ*_E_ of the respiratory system *excluding* the endotracheal tube and ventilator apparatus using *P*_TR_. To allow the assessment of *τ*_E_ heterogeneity, the *V*_E_ from the point of maximum $\dot{V}$_E_ to the end of expiration (defined as $\dot{V}$_E_ less than 0.05 L/sec) for each ensemble was divided into five equal volume segments (*V*_E1_ − *V*_E5_). We chose to use five segments in keeping with methods from similar studies (Guttmann et al. 1995; Kondili et al. 2002). Each of the five *V*_E_ segments was assumed to have *E* and *R* values that did not vary throughout the duration of the segment. Therefore for each *V*_E_ segment, the *τ*_E_ was calculated as the quotient of *R*_RS_ and *E*_RS_. *R*_RS_ was calculated as (*P*_TR_ − *P*_ATM_)/ $\dot{V}$_E_ and *E*_RS_ as ∆*P*_TR_/∆*V*_E_ for the *V*_E_ segment in question. This method allowed the calculation of unique respiratory system time constants for each of the five *V*_E_ segments (named *τ*_E1_ through *τ*_E5_) from the point of maximum $\dot{V}$_E_ to end expiration (Fig. 1).

### Statistical analyses and model

Descriptive statistics are displayed as mean ± standard deviation. Other values are displayed as mean and 95% confidence intervals (CI). Continuous variables were analyzed using paired *t* tests (within animal) or two‐sample *t* tests (between animals), where appropriate. All tests were two‐sided and the statistical significance was defined at *P* < 0.05. Statistical analyses were performed using STATA 10.0 Statistical Software (StataCorp, College Station, TX) and SAS (SAS Institute, Inc., NC).

Expired time constants values were analyzed by linear mixed‐effect model, including a random effect for each animal and fixed effects for segment, PEEP, *V*_T_, and pre–post injury.

Significance of model coefficient estimates, least squares means, and differences in least squares means were determined by *T* test. Main effects and interactions were confirmed by use of *F* tests with type III sums of squares. All tests were performed at the 0.05 significance level. Differences in least squares means were adjusted for multiple testing using the Tukey–Kramer adjustment.

## Results

Before lung injury, the animals demonstrated a PaO_2_ of 197 ± 51 mmHg while ventilated with a *V*_T_ of 10 cc/kg, 0 cm H_2_O PEEP and an FiO_2_ of 0.5. Thirty minutes after injury, PaO_2_ decreased to 68 ± 10 mmHg and was 69 ± 19 mmHg prior to euthanasia (*P *<* *0.01 for both time points compared to preinjury values). During the same preinjury ventilation conditions, FRC was 14.3 ± 2.5 mL/kg and decreased to 9 ± 2.9 mL/kg after injury (*P *<* *0.01). *E*_RS_ and *R*_RS_ both increased with experimental injury due to significant increases in both *E*_L_ and *R*_L_ (*P *<* *0.01 for both) and no changes in *E*_CW_ or *R*_CW_ (*P *>* *0.05). Descriptive pulmonary mechanical data are presented in Table 1.

**Table 1:** Pulmonary mechanical data before and after experimental lung injury

	*E*_RS_ (cm H_2_O/L)	*R*_RS_ (cm H_2_O/L/sec)	*E*_L_ (cm H_2_O/L)	*R*_L_ (cm H_2_O/L/sec)	*E*_CW_ (cm H_2_O/L)	*R*_CW_ (cm H_2_O/L/sec)
Before injury	37.6 ± 11.9	7.1 ± 1.6	25.5 ± 10.0	6.1 ± 1.6	12.1 ± 4.7	1.0 ± 0.3
After injury	65.6 ± 27.6	18.2 ± 5.7	55.4 ± 27.8	16.9 ± 5.4	10.2 ± 3.3	1.3 ± 1.0
*P* value	0.010	0.0026	0.011	0.0024	0.16	0.29

*E*_RS_, elastance of the respiratory system; *E*_L_, elastance of the lung; *E*_CW_, elastance of the chest wall; *R*_RS_, resistance of the respiratory system; *R*_L_, resistance of the lung; *R*_CW_, resistance of the chest wall. Elastance data for this table were collected during end‐inspiratory plateau conditions.

All data are presented as mean and 95% confidence intervals. *P* value compares the before and after injury values.

### Effect of injury status, PEEP, and tidal volume on expiratory time constants

**Figure 2:**
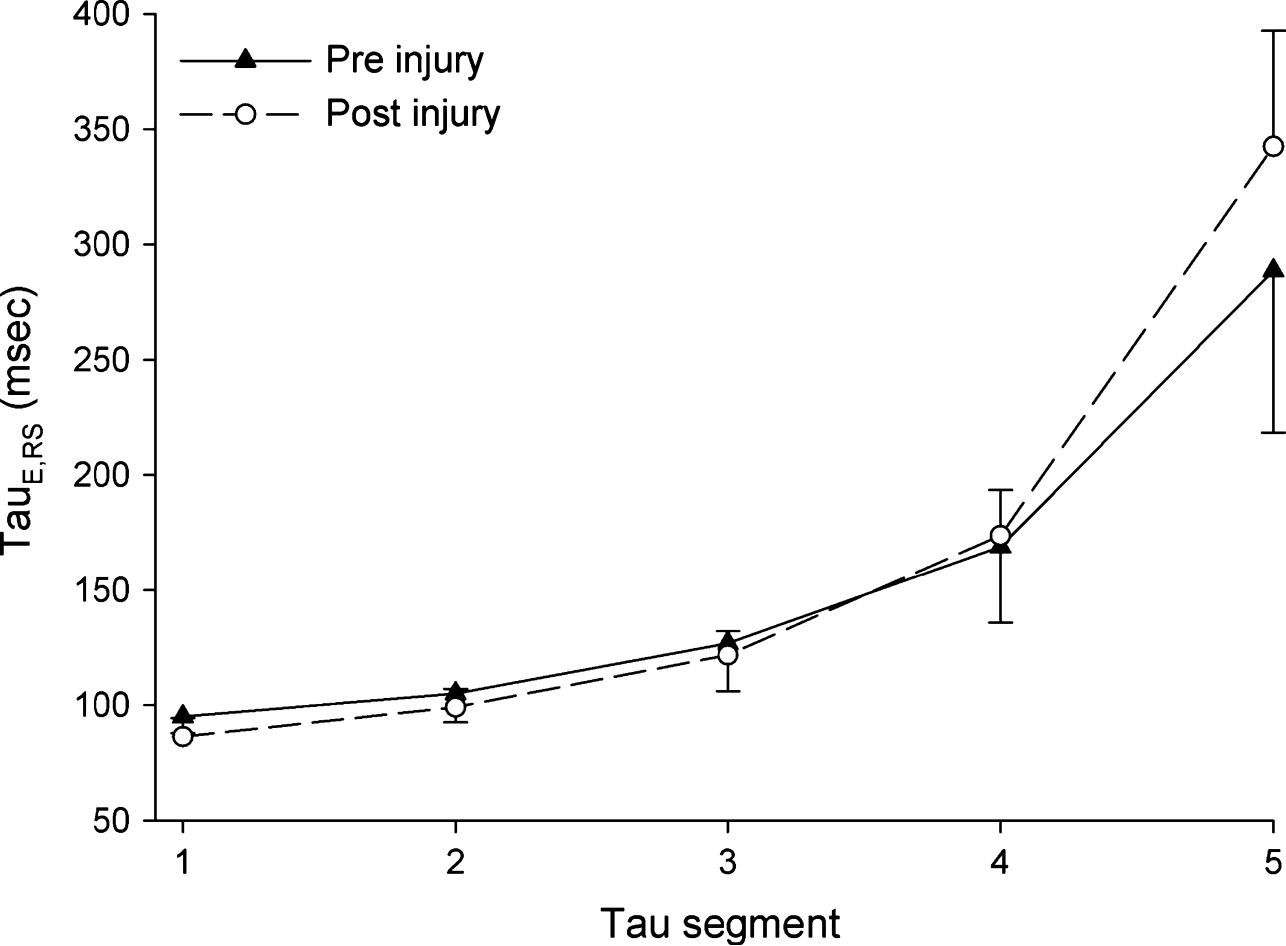
Values for *τ*_E,_ _RS__1_ to *τ*_E,_ _RS__5_ before and after injury are displayed as mean values ± 95% confidence interval.

Under all conditions of PEEP and *V*_T_, *τ*_E_ increased throughout expiration both before and after injury (Table 2 and Fig. 2). Segmented *τ*_E_ values increased throughout expiration with a slope that was different than zero (*P *<* *0.01). The expired time constant increased by an average of 45.08 msec per segment when *τ*_E_ segment was treated as a continuous variable (36.07, 54.08; 95% CI). The model used for this analysis included an interaction between segment and injury. The main effect of segment as a continuous covariate was significant with and without interaction terms. Congruent with these findings, *τ*_E1_ and *τ*_E2_ were significantly smaller than *τ*_E4_ and *τ*_E5_, *τ*_E3_ showed significant difference from *τ*_E4_ and *τ*_E5_, and there was a difference between *τ*_E4_ and *τ*_E5_ (*P *<* *0.01 for all comparisons). When an interaction between injury status and *τ*_E_ segment was included in the model, it was found to be significant (*P *<* *0.05), indicating that later segments (*τ*_E4_ and *τ*_E5_) had higher values post injury compared to before injury.

**Table 2:** Effect of injury by expired volume segment on *τ*_E_

	*τ*_E,RS1_ (msec)	*τ*_E,RS2_ (msec)	*τ*_E,RS3_ (msec)	*τ*_E,RS4_ (msec)	*τ*_E,RS5_ (msec)
Before injury	95.1 (46.6, 143.7)	105.1 (56.5, 153.7)	126.9 (78.3, 175.4)	168.9 (120.4, 217.5)	288.6 (240.1, 337.2)
After injury	86.4 (37.9, 135.0)	99.1 (50.6, 147.7)	121.8 (73.2, 170.3)	173.7 (125.2, 222.3)	342.4 (298.9, 391.0)
Difference	−8.7 (−67.8, 50.4)	−6.0 (−65.1, 53.1)	−5.1 (−64.2, 53.9)	4.8 (−54.2, 63.9)	53.8 (−5.3, 112.9)

*τ*_E,RS1–5_, expiratory time constant for expired volume slice 1 through 5; msec, milliseconds. All data are presented as mean and 95% confidence intervals.

**Figure 3:**
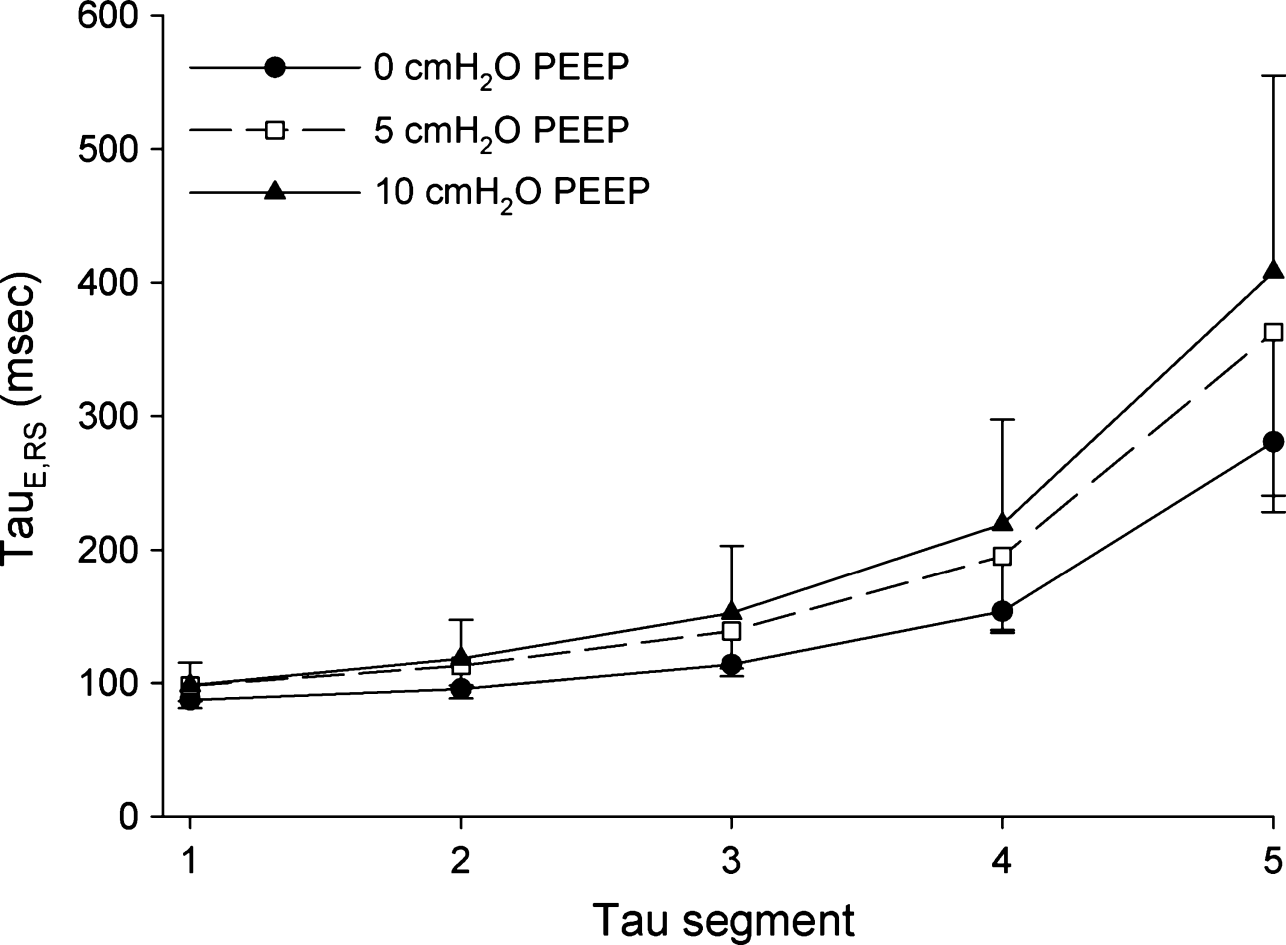
Values for *τ*_E,_ _RS__1_ to *τ*_E,_ _RS__5_ at each positive end‐expiratory pressure (PEEP) setting are displayed as mean values ± 95% confidence interval.

**Figure 4:**
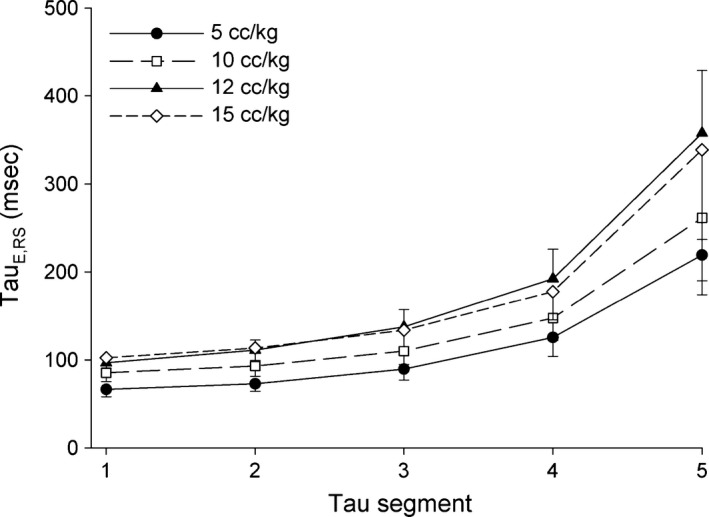
Values for *τ*_E,_ _RS__1_ to *τ*_E,_ _RS__5_ _RS__5_ at each tidal volume (*V*_T_) setting are displayed as mean values ± 95% confidence interval.

Higher PEEP and *V*_T_ values were associated with higher *τ*_E_ values (Figs. 3 and 4). The increase in *τ*_E_ per 1 mL/kg increase in *V*_T_ and 1 cm H_2_O increase in PEEP and were 5.97 msec (3.23, 8.70; 95% CI) and 4.53 msec (2.34, 6.72; 95% CI), respectively. No evidence was found for an interaction between injury status and *V*_T_, or between injury status and PEEP. The raw segment values for *R*_RS_, *E*_RS_, and *τ*_E_ are shown in [Link].

### Effect of injury status, PEEP, and tidal volume on resistance and elastance

To further clarify the causative factors behind changes in *τ*_E_ segments throughout expiration, we analyzed *R*_RS_ and *E*_RS_ on a per segment basis using the same methods applied to *τ*_E_ segments *τ*_E1_ to *τ*_E5_. Both *R*_RS_ and *E*_RS_ were significantly increased after injury compared to before injury in early *V*_E_ segments (Tables 3 and 4). Segment values for *R*_RS_ and *E*_RS_ after injury were statistically similar to values before injury (*P *>* *0.05).

**Table 3:** Effect of injury by expired volume segment on *R*_RS_

	*R*_RS1_ (cm H_2_O/L/sec)	*R*_RS2_ (cm H_2_O/L/sec)	*R*_RS3_ (cm H_2_O/L/sec)	*R*_RS4_ (cm H_2_O/L/sec)	*R*_RS5_ (cm H_2_O/L/sec)
Before injury	5.3 (3.6, 7.0)	4.6 (2.9, 6.4)	4.4 (2.7, 6.1)	4.8 (3.0, 6.5)	7.4 (5.7, 9.1)
After injury	14.3 (12.6, 16.1)	9.4 (7.7, 11.2)	7.0 (5.3, 8.7)	6.9 (5.1, 8.6)	8.5 (6.7, 10.2)
Difference	9.1 (5.9, 12.2)	4.8 (1.7, 7.9)	2.6 (−0.5, 5.7)	2.1 (−1.0, 5.2)	1.1 (−2.0, 4.2)

*R*_RS1–5_, respiratory system resistance for expired volume slice 1 through 5. All data are presented as mean and 95% confidence intervals.

**Table 4:** Effect of injury by expired volume segment on *E*_RS_

	*E*_RS1_ (cm H_2_O/L)	*E*_RS2_ (cm H_2_O/L)	*E*_RS3_ (cm H_2_O/L)	*E*_RS4_ (cm H_2_O/L)	*E*_RS5_ (cm H_2_O/L)
Before injury	54.1 (39.6, 68.5)	45.1 (30.6, 59.5)	36.5 (22.0, 50.9)	30.5 (16.0, 44.9)	27.6 (13.1, 42.0)
After injury	159.8 (145.4, 174.3)	96.1 (81.7, 110.6)	60.6 (46.1, 75.0)	42.8 (28.4, 57.3)	27.5 (13.0, 41.9)
Difference	105.8 (82.3, 129.2)	51.0 (27.6, 74.5)	24.1 (0.6, 47.5)	12.3 (−11.1, 35.8)	0.1 (−23.4, 23.5)

*E*_RS1–5_, elastance for expired volume slice 1 through 5. All data are presented as mean and 95% confidence intervals.

The differing values for *R*_RS_ and *E*_RS_ between pre‐ and post injury status was confirmed by the presence of a significant interaction effect between injury status and segment (*P *<* *0.01). The effect of segment on *R*_RS_ as a continuous variable was not significant. However, segment as a categorical covariate was significant (*P *<* *0.01) in determining the value of *R*_RS_. The estimated slope of *E*_RS_ by segment was −6.76 (−10.14, −3.38; 95% CI) and was significant (*P *<* *0.01). Segment remained a significant continuous covariate when included in the model for *E*_RS_ without an interaction term. No evidence of other interactions was found.

**Figure 5:**
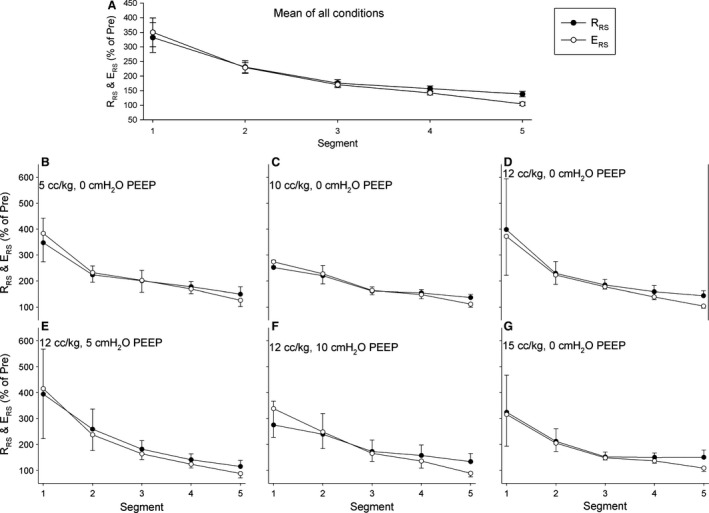
Relative values (comparing postinjury to preinjury state) for *R*_RS_ and *E*_RS_ for each equivolemic segment. Values are displayed as the mean of all positive end‐expiratory pressure (PEEP) and tidal volume (*V*_T_) values (A), and for individual combinations of PEEP and *V*_T_ (B–G).

Relative values (comparing postinjury to preinjury state) for *R*_RS_ and *E*_RS_ for each volume segment are shown in Figure 5, while raw values are shown in [Link]. Over both states of injury, *R*_RS1_ is significantly different from all subsequent segments except for *R*_RS5_ (*P *<* *0.01), as is the difference between *R*_RS5_ and *R*_RS1_ − *R*_RS4_ (*P *<* *0.01). However, the differences in *R*_RS_ by segment may have been driven by postinjury values, as no significant differences were found between *R*_RS_ segments preinjury. Post injury, *R*_RS1_ differs from all other segments post injury (*P* < 0.01). For *E*_RS_, *E*_RS1_ and *E*_RS2_ were significantly different from *E*_RS3_, *E*_RS4_, and *E*_RS5_ (*P* < 0.01), as is the *E*_RS3_ from *E*_RS4_ and *E*_RS5_ (*P* < 0.01). Within injury state, postinjury differences again outnumbered the preinjury segment differences; Post injury, *E*_RS1_ is different from all others (*P* < 0.01), *E*_RS2_ is significantly different from *E*_RS3_, *E*_RS4_, and *E*_RS5_ (*P* < 0.01), and *E*_RS3_ and *E*_RS5_ are different (*P* < 0.01). Pre injury, only *E*_RS1_ is significantly different from *E*_RS4_ and *E*_RS5_ (*P* < 0.05).

Finally, we found that changes in PEEP or *V*_T_ altered *R*_RS_ and *E*_RS_. A 1 cm H_2_O increase in PEEP increased *R*_RS_ by 0.21 cm H_2_O/L/sec (0.09, 0.33; 95% CI), and increased *E*_RS_ by 1.21 cm H_2_O/L (0.34, 2.08; 95% CI). A 1 mL/kg increase in *V*_T_ increased *R*_RS_ by 0.24 cm H_2_O/L/sec (0.09, 0.38; 95% CI), but did not have any significant effect on *E*_RS_.

## Discussion

The current study has three main findings. First, consistent with our previous work (Henderson et al. 2015), *τ*_E_ increased throughout expiration, and injury increased the difference between late and early *τ*_E_ segments compared to before injury. These changes were due to increases in both *R*_RS_ and *E*_RS_. Second, we found that manipulating PEEP or *V*_T_ did not have a differential effect on *τ*_E_ between injured and uninjured states in a segmented model of expiration. Third, increases in both PEEP and *V*_T_ increased *R*_RS_, a finding that has potential clinical implications for the choice of mechanical ventilation strategy in patients with ARDS or lung injury.

### Injury and time constants

Using a single‐compartment model rather than a segmented model, we observed that injury decreased average *τ*_E_ values compared with before injury (Table 2). The decreased *τ*_E_ observed in the postinjury state is related to a relative decrease in *R*_RS_ compared to *E*_RS_. Following lung injury, we found that increases in *R*_RS_ and *E*_RS_ were driven by increases in *R*_L_ and *E*_L_ with no significant changes in *R*_CW_ and *E*_CW_. Accordingly, the absence of mechanical alterations to the chest wall suggests that the injury used in this model was confined to the lungs (Table 1). When assessed from the perspective of a single compartment (i.e., not segmented), increases in *E*_L_ were greater than those in *R*_L_ and therefore *τ*_E_ decreased after injury.

Many methods of calculating *τ*_E_ assume that all lung units inflate and deflate as a single compartment and are thus unable to distinguish between units that fill/empty quickly and units that require greater time. We therefore employed a segmental method that can be used to more accurately describe the pattern of lung emptying during passive expiration, potentially providing more insight into the degree of pulmonary mechanical heterogeneity than is afforded by a single‐compartment model (Guttmann et al. 1995; Lourens et al. 2000; Kondili et al. 2002, 2004; Henderson et al. 2015). When a segmented model was used to analyze our data, we were able to observe the mechanical properties of passive expiration with a higher degree of resolution than is afforded by the single‐compartment model. In a segmented model, injury increased the difference between later versus earlier segment *τ*_E_ values – that is, injury decreased the average *τ*_E_ in early segments, and increased the average *τ*_E_ for later segments compared to the uninjured state (Table 2 and Fig. 2).

When compared with uninjured values, both *R*_RS_ and *E*_RS_ after injury are roughly threefold higher at the beginning of expiration (Fig. 5A) before returning to values similar to those in uninjured lungs by the end of expiration. While both *R*_RS_ and *E*_RS_ increased postinjury, the increase in *E*_RS_ in absolute terms was greater, causing early *τ*_E_ values (e.g., *τ*_E1_ in Table 2) to be smaller after injury compared to before injury. Throughout expiration, values of *E*_RS_ decreased more rapidly than *R*_RS_, causing late *τ*_E_ segment values to be higher (e.g., *τ*_E5_ in Table 2).

### PEEP and tidal volume

We found that increasing both PEEP and *V*_T_ increased *τ*_E_, but that these effects did not differ between the uninjured and injured states (Figs. 3 and 4). We demonstrated that increases in PEEP increased *R*_RS_ and increased *E*_RS_, while increases in *V*_T_ increased *R*_RS_ alone. The independent effects of PEEP and *V*_T_ on *τ*_E_ segments appeared consistent throughout expiration, that is, they were not confined to early or late *τ*_E_ segments.

It is instructive to compare our results with those of other investigators. Kondili and colleagues reported effects of PEEP that differed from ours (Kondili et al. 2002). They found that in subjects with ARDS who were ventilated without PEEP, *τ*_E_ and *R*_RS_ increased significantly in late expiration and that the addition of PEEP eliminated these findings. One result of the application of PEEP is that small airways are “splinted” open, and are less likely to close prematurely. The increased patency of small airways may allow more rapid exhalation, thereby resulting in smaller *τ*_E_ values with higher PEEP, particularly in late expiration. This widely accepted concept supplies a satisfying explanation for the effect of PEEP in Kondili's results. However, other authors have found patterns of expiration and effects of applied PEEP that are more similar to our results. Pesenti and colleagues found that PEEP increased *R*_RS_ in patients with ARDS and in normal controls (Pesenti et al. 1991). In patients with ARDS, Mols and colleagues observed that the pattern of *τ*_E1–5_ and R_RS1–5_ was highly variable, with patients demonstrating steady increases, steady declines, or no discernible pattern during passive expiration (Mols et al. 2001). Similarly, other authors have demonstrated that increases in PEEP and *V*_T_ increase *R*_RS_ in subjects with lung injury or ARDS (Auler et al. 1990; Tantucci et al. 1992; Pelosi et al. 1995). Current management of ARDS often involves the use of high PEEP levels. In general, this improves oxygenation and may decrease new ventilator‐associated lung injury (Gattinoni et al. 2012). However, our data suggest that increased PEEP or *V*_T_ may prolong expiration due to increased emptying time for lung units with long time constants. This may be of relevance in patients who develop ARDS in the context of lung disease that predisposes them to long time constants or gas trapping, such as COPD or asthma. In this subset of patients, a more conservative PEEP strategy may be warranted.

There are several possible explanations for the finding that increases in PEEP or *V*_T_ may increase *R*_RS_ First, injured lung is characterized by inhomogeneity of distension, and high levels of PEEP could further overdistend some units, thereby increasing time constant inhomogeneity. Second, increased *R*_RS_ at higher PEEP or *V*_T_ values may in part be due to stress adaptation phenomena and specifically increases in viscoelastic resistance at higher PEEP values and lung volumes (Pesenti et al. 1991; Pelosi et al. 1995). Third, it has been suggested that the longitudinal stretching of airways at high PEEP and *V*_T_ levels may narrow their cross‐sectional area and thus increase resistance to gas flow (Eissa et al. 1991a). Given these data, observation of the pattern of *τ*_E_ during expiration may facilitate the choice of less injurious strategies of mechanical ventilation during the care of patients with ARDS or lung injury.

### Limitations

The current experiment reveals novel findings regarding the relationships between lung injury, the parameters of mechanical ventilation, and the pattern of $\dot{V}$_E._ However, several limitations must be considered. First, we did not measure *P*_TR_ directly in this experiment. Instead, like Guttmann, we estimated *P*_TR_ from *P*_AW_ using a previously validated method (Guttmann et al. 1993, 1995; Zhao et al. 2010). Guttmann and colleagues measured *P*_AW_ at the ventilator wye and observed that the calculated *τ*_E_ for sequential volume segments in mechanically ventilated ARDS patients were almost identical throughout expiration (Guttmann et al. 1995). The similar *τ*_E_ was due to the significant flow‐dependent resistance of the endotracheal tube and ventilator (*R*_AW_) (Wright and Bernard 1989; Guttmann et al. 1993). Given that flow is greatest at the onset of passive expiration, the relative contribution of *R*_AW_ on *τ*_E_ is higher during early expiration and lower in late expiration, which masks the influence of *R*_L_ (Wright and Bernard 1989; Guttmann et al. 1993). When they eliminated the effect of the endotracheal tube by estimating *P*_TR_ and used these values to calculate segmental values for *τ*_E_, Guttman and colleagues found a pattern of steadily increasing *τ*_E_ throughout expiration. Similarly, our data demonstrate that *τ*_E_ increases steadily throughout expiration when a segmented model that excludes the resistance of the endotracheal tube was used (Fig. 2). However, some caution is warranted when using the model of endotracheal tube resistance compensation such as that used in this experiment. Clinical patients with ARDS may have secretions that can alter endotracheal tube resistance in ways that are not modeled by benchtop testing.

## Conclusions

Our study provides several novel insights into the details of expiratory gas flow in animals before and after experimental lung injury. It has previously been observed that *τ*_E_ values are smaller in subjects with injured lungs when compared to controls. However, we are the first to demonstrate this change within subjects and that *τ*_E_ increased throughout expiration both before and after injury when examined with a multiple compartment model. Finally, we have demonstrated that increases in PEEP or *V*_T_ increased *τ*_E_ throughout expiration, but did not appear to have effects that differed between the uninjured and injured state. Whether incorporating the pattern of *τ*_E_ will improve strategies of mechanical ventilation in lung injury and ARDS needs to be assessed in future studies.

## Conflict of Interest

None declared.

## Raw values for selected pulmonary mechanical variables by expired volume segment.

**Table:**

*V*_T_	PEEP (cm H_2_O)	Volume Segment	Pre			Post		
			*R*_RS_ (cm H_2_O/L/sec)	*E*_RS_ (cm H_2_O/L)	*τ*_E_ (msec)	*R*_RS_ (cm H_2_O/L/sec)	*E*_RS_ (cm H_2_O/L)	*τ*_E_ (msec)
5 mL/kg	0	1	2.80 ± 0.78	41.02 ± 12.76	70.39 ± 12.51	9.25 ± 4.48	143.01 ± 60.48	62.76 ± 11.61
		2	2.93 ± 0.67	39.58 ± 8.56	75.49 ± 13.32	6.28 ± 2.20	90.55 ± 33.48	70.29 ± 11.82
		3	3.45 ± 1.01	38.42 ± 8.20	91.35 ± 19.74	6.35 ± 2.34	74.13 ± 27.15	87.62 ± 16.56
		4	4.01 ± 0.86	34.15 ± 8.08	124.90 ± 38.10	7.00 ± 1.92	58.07 ± 18.76	126.16 ± 24.62
		5	5.47 ± 1.78	28.79 ± 9.36	203.46 ± 75.87	7.37 ± 2.09	33.58 ± 11.70	235.24 ± 53.06
10 mL/kg	0	1	3.97 ± 1.14	43.59 ± 9.64	90.02 ± 14.92	9.65 ± 2.68	116.61 ± 16.97	80.98 ± 13.73
		2	3.65 ± 0.87	39.42 ± 9.94	94.65 ± 17.13	7.51 ± 1.78	85.30 ± 23.93	91.18 ± 16.95
		3	3.99 ± 0.73	36.90 ± 5.81	110.96 ± 23.21	6.24 ± 0.89	60.46 ± 13.97	108.93 ± 21.88
		4	4.34 ± 0.89	31.50 ± 6.15	144.29 ± 39.92	6.47 ± 0.81	45.90 ± 10.33	150.71 ± 35.35
		5	6.31 ± 2.13	28.98 ± 5.92	238.36 ± 107.91	8.16 ± 1.82	31.82 ± 7.39	284.91 ± 100.36
12 mL/kg	0	1	4.79 ± 1.52	49.16 ± 14.25	96.79 ± 14.43	14.14 ± 7.69	149.95 ± 71.98	91.99 ± 13.97
		2	4.33 ± 1.30	43.62 ± 15.65	103.16 ± 18.40	9.21 ± 3.12	91.07 ± 27.21	101.98 ± 16.63
		3	3.89 ± 0.66	33.10 ± 6.27	121.45 ± 25.22	6.95 ± 1.09	59.02 ± 12.42	121.86 ± 18.84
		4	4.60 ± 1.20	30.53 ± 6.70	156.62 ± 44.02	6.81 ± 1.05	41.89 ± 8.06	169.40 ± 32.67
		5	7.02 ± 3.12	28.20 ± 6.36	266.85 ± 127.02	9.21 ± 2.85	28.92 ± 5.94	338.55 ± 114.55
	5	1	5.89 ± 2.06	57.53 ± 19.26	101.23 ± 13.41	17.02 ± 8.87	176.77 ± 78.07	94.50 ± 19.94
		2	4.99 ± 1.39	45.84 ± 14.11	113.16 ± 27.05	10.85 ± 3.48	98.40 ± 31.72	112.23 ± 13.84
		3	4.45 ± 1.39	34.34 ± 8.30	138.47 ± 53.03	7.33 ± 1.70	54.73 ± 14.47	138.95 ± 25.55
		4	5.24 ± 2.29	29.66 ± 6.76	195.02 ± 106.88	6.53 ± 1.39	35.94 ± 9.40	194.97 ± 55.95
		5	10.97 ± 8.69	30.06 ± 9.71	359.14 ± 252.48	7.75 ± 2.66	22.59 ± 5.16	366.49 ± 126.25
	10	1	8.05 ± 2.39	75.98 ± 21.50	106.19 ± 22.35	18.52 ± 10.52	211.11 ± 84.45	89.93 ± 26.05
		2	6.87 ± 2.26	56.84 ± 15.54	129.55 ± 57.07	12.95 ± 4.26	122.80 ± 36.86	106.62 ± 16.74
		3	6.08 ± 2.43	41.77 ± 11.06	165.48 ± 102.51	8.71 ± 2.10	64.25 ± 17.74	139.31 ± 20.17
		4	5.81 ± 3.13	29.11 ± 6.42	221.92 ± 150.90	7.75 ± 3.05	36.93 ± 11.97	216.26 ± 64.67
		5	7.31 ± 4.39	22.53 ± 4.86	375.12 ± 271.56	8.39 ± 3.98	19.05 ± 5.50	440.72 ± 139.74
15 mL/kg	0	1	6.17 ± 2.01	57.13 ± 14.99	106.23 ± 15.33	17.41 ± 13.24	162.55 ± 100.61	98.47 ± 19.98
		2	5.06 ± 1.59	45.10 ± 14.08	114.58 ± 20.68	9.80 ± 3.24	88.55 ± 26.18	112.34 ± 21.17
		3	4.42 ± 0.77	34.44 ± 6.90	133.52 ± 27.92	6.50 ± 1.07	50.90 ± 10.48	133.88 ± 28.33
		4	4.57 ± 0.84	27.95 ± 4.40	170.75 ± 46.66	6.64 ± 1.03	38.17 ± 7.34	184.88 ± 48.11
		5	7.24 ± 2.80	26.82 ± 5.27	288.82 ± 134.81	9.95 ± 3.11	29.07 ± 7.90	388.69 ± 153.55
